# Identifying Best Implementation Practices for Smoking Cessation in Complex Cancer Settings

**DOI:** 10.3390/curroncol28010049

**Published:** 2021-01-13

**Authors:** Eleni Giannopoulos, Janet Papadakos, Erin Cameron, Janette Brual, Rebecca Truscott, William K. Evans, Meredith Elana Giuliani

**Affiliations:** 1 Cancer Education Program, Princess Margaret Cancer Centre, Toronto, ON M5G 2C4, Canada; eleni.giannopoulos@uhnresearch.ca (E.G.); janet.papadakos@uhnresearch.ca (J.P.); janette.brual@uhnresearch.ca (J.B.); 2 Patient Education, Ontario Health (Cancer Care Ontario), Toronto, ON M5G 2L3, Canada; 3 Institute for Health Policy, Management & Evaluation, University of Toronto, Toronto, ON M5T 1P8, Canada; 4 Prevention & Cancer Control, Ontario Health (Cancer Care Ontario), Toronto, ON M5G 1X3, Canada; erin.cameron@cancercare.on.ca (E.C.); rebecca.truscott@cancercare.on.ca (R.T.); 5 Department of Oncology, McMaster University, Hamilton, ON L8S 4L8, Canada; billevans@cogeco.ca; 6 Radiation Medicine Program and Cancer Education Program, Princess Margaret Cancer Centre, Toronto, ON M5G 2M9, Canada; 7 Department of Radiation Oncology, University of Toronto, Toronto, ON M5T 1P5, Canada

**Keywords:** quality improvement, smoking cessation, cancer, intervention, implementation, realist evaluation

## Abstract

Background: In response to evidence about the health benefits of smoking cessation at time of cancer diagnosis, Ontario Health (Cancer Care Ontario) (OH-CCO) instructed Regional Cancer Centres (RCC) to implement smoking cessation interventions (SCI). RCCs were given flexibility to implement SCIs according to their context but were required to screen new patients for tobacco status, advise patients about the importance of quitting, and refer patients to cessation supports. The purpose of this evaluation was to identify practices that influenced successful implementation across RCCs. Methods: A realist evaluation approach was employed. Realist evaluations examine how underlying processes of an intervention (mechanisms) in specific settings (contexts) interact to produce results (outcomes). A realist evaluation may thus help to generate an understanding of what may or may not work across contexts. Results: The RCCs with the highest Tobacco Screening Rates used a centralized system. Regarding the process for advising and referring, three RCCs offered robust smoking cessation training, resulting in advice and referral rates between 80% and 100%. Five RCCs surpassed the target for Accepted Referral Rates; acceptance rates for internal referral were highest overall. Conclusion: Findings highlight factors that may influence successful SCI implementation.

## 1. Introduction

Considerable evidence demonstrates that smoking cessation (SC) results in improved health outcomes for cancer patients. This includes reduced overall and cancer-specific mortality, improved treatment efficacy, decreased treatment toxicities and decreased risk of cancer recurrence [1,2,3]. A cancer diagnosis serves as an opportunity for healthcare providers to intervene and help patients quit smoking [4]. While several clinical practice guidelines exist to help organizations implement evidence-based SC interventions [5], many healthcare providers do not adequately utilize these interventions in practice [6]. In 2012, Ontario Health (Cancer Care Ontario) (OH-CCO)-the agency responsible for overseeing cancer programs and health services in Ontario, Canada—instructed all Regional Cancer Centres (RCCs) to implement a SC intervention [7]. The intervention included the mandatory screening of new ambulatory cancer patients for smoking status, advising current or recent smokers about the benefits of quitting and providing a referral to a SC service. The RCCs were able to decide how to implement the intervention and tailor it to their local context. Given that implementing a SC intervention is a complex undertaking, we used a realist evaluation approach to identify variables that contribute to successful implementation in diverse environmental settings, and ultimately improve integration of the intervention in practice. 

### Background

Ontario consists of 14 geographic regions, each of which has a RCC and a network of hospitals and other agencies responsible for providing cancer services [8,9]. The 14 regions vary in population size and density, socio-demographics and geographic range [7].

OH-CCO developed the Framework for Smoking Cessation to guide implementation of SC interventions in the RCCs. The Framework for Smoking Cessation consisted of three parts [7]. The first part described the standard elements that each intervention must include. This included: designating a SC “champion” to lead implementation; implementing the SC intervention (screen, advise, refer); staff training; and regular reporting to OH-CCO. The second part described options that could vary across RCCs. This included the: option for intervention intensity (brief or intensive) [5]; the type of referral (internally or externally provided); links to community supports; and strategies to promote the intervention. The third part was the establishment of an OH-CCO centralized support system database for analytics and reporting. 

Although organizations are often required to implement new policy changes and evidence-based practices, many of these practices are not sustained [10]. The literature has cited several factors that influence implementation of hospital-based interventions. These include barriers and facilitators, such as staff awareness and attitudes, seamless integration of the intervention in practice and organizational culture and environment [11]. Further, there is a dearth of studies examining implementation barriers to, and facilitators of, hospital-based SC interventions in a cancer setting. In this unique approach, we employed a realist evaluation methodology to understand how each RCC implemented OH-CCO’s SC intervention across Ontario, and offer insight into why RCCs had variable success.

We first sought to compare outcomes between RCCs that implemented the SC intervention. We then explored how components related to the organizational context and intervention mechanism interacted to produce the observed outcomes. Though all RCCs were required to implement the SC intervention, the approach they used varied. An understanding of how the interventions were implemented in different RCCs can offer insight into why successful outcomes were seen in some RCCs but not in others [12]. As such, the purpose of this evaluation was to: (1) determine how each RCC implemented the SC intervention; (2) learn about the conditions that enable successful implementation within specific contexts; and (3) identify a set of best practices to support and improve integration of the intervention in diverse settings. We conducted the evaluation four years following the launch of OH-CCO’s Framework for Smoking Cessation to allow RCCs the opportunity to implement the intervention and refine their practices, such that findings from the evaluation would be most helpful.

## 2. Methods

### 2.1. Quality Improvement: Realist Evaluation

We conducted a Quality Improvement (QI) evaluation, using a realist approach, to evaluate the implementation of SC interventions at each RCC. This is a context-focused methodology that can help guide the adoption of interventions in dynamic and complex environments, and help inform best practice [13,14,15,16]. The realist evaluation examines underlying processes of an intervention (mechanisms) in specific settings (contexts). The interaction between these two variables generates specific results (outcomes) [17,18,19]. In other words, the mechanism is what allows the intervention to operate, but may produce varying outcomes depending on the setting [20]. Hence, a realist approach may help generate an understanding of what may or may not work in different contexts. As such, evaluating and reporting on implementation practices in fourteen unique cancer settings may assist others across the globe who wish to implement SC interventions within their local contexts. 

### 2.2. Programme Theory

Realist evaluations begin with a program theory. OH-CCO’s requirement for RCCs to implement SC interventions was based on substantial evidence that SC has significant benefits to individuals that have cancer, as well as to the health system [21]. OH-CCO allowed RCCs to be flexible in their approach, in hopes that tailoring the intervention would produce successful results in each context. The programme theory for this evaluation was that it is possible to extend learning from specific contexts to promote successful implementation practices.

### 2.3. Variables

The QI team determined the Context-Mechanism-Outcome (CMO) variables a priori. Context and Mechanism variables consisted of factors that could influence implementation, while Outcomes consisted of the OH-CCO performance indicators.

Identified variables were coded as “context” if they were considered to be conditions that influenced the functionality of the intervention mechanism [22] (e.g., Cancer Centre Size or Smoking Cessation Education). Variables were coded as “mechanism” if they were considered to be processes or resources that explained how the intervention operated [22] (e.g., Patient Flow Process for Tobacco Screening). Identified variables were coded as “outcomes”, as they were considered to be the result of the intervention [22] (e.g., Tobacco Screening Rates). In addition to the variables we identified, we observed specific patterns in the data and formed subsequent context-mechanism-outcome linkages [23]. The linkages provided a framework for data interpretation. Triangulation of various sources of data was used to strengthen interpretation of data and conclusions [24].

Context variables were the Cancer Centre Size, Smoking Cessation Education, Smoking Cessation Model and Referral System. Mechanism variables were the Patient Flow Process for Tobacco Screening, the Patient Flow Process for Advising and Referring, and Pharmacotherapy.

We used data from the five performance indicators for SC, as established by OH-CCO, to describe intervention outcomes. Indicators included the proportion of: (i) ambulatory cancer patients screened for tobacco use; (ii) current (or recent) smokers; (iii) smokers advised on the benefits of cessation; (iv) smokers recommended for referral; (v) smokers who accepted a referral. The type of referral was also determined to be either internal (within the RCC), external (another health care facility, agency, or helpline) or a combination of both. OH-CCO targets were set for two performance indicators: 70% for new ambulatory cancer patients screened for tobacco use and 20% for smokers who accepted a referral to a cessation service.

### 2.4. Data Collection and Analysis

Components of the realist evaluation methodology guided data collection and analysis [23]. A realist evaluation begins with an exploration of data using a mixed-methods approach, followed by analysis to help construct context–mechanism–outcome linkages [19,22]. All data collection methods were designed to collect CMO variables.

As part of the QI evaluation, fourteen semi-structured telephone interviews were conducted with SC champions across RCCs to gain an understanding of how the SC intervention was implemented. Team members developed the interview guide with input from an advisory committee of experts. Open-ended questions were used to elicit thoughtful discussion. Half-day site visits were conducted to complement the interviews and to observe the intervention. Site visits included: a tour of the RCC led by the SC champion; observation of frontline staff screening (e.g., nurses, clerical staff), advising and referring patients; and a review of SC data collection practices. SC champions came from a variety of backgrounds (e.g., smoking cessation counsellor, radiation oncologist, and dentist). Data from the semi-structured interviews and field notes from the site visits were coded and themes were identified. Themes were sorted into the “context” or “mechanism” category as they related to implementation of the SC intervention. OH-CCO’s performance indicators constituted the “outcomes” of the SC intervention.

Interviews were audio-recorded, transcribed verbatim, and reviewed against notes from site visits. A coding structure was determined a priori. Interview transcripts were reviewed and coded by one team member through a manual, iterative process. A second team member reviewed the coding and there was no disagreement between coders. After the data was coded, similar codes were merged. Indicator data, extracted from OH-CCO’s database, was analyzed to produce aggregate outcome variables.

Using the context-mechanism-outcome framework as a guide, two team members coded the variables from the data under the context, mechanism and outcome categories (Table 1). A third team member reviewed the coding to adjudicate any disagreement.

### 2.5. Ethical Considerations

The University Health Network Research Ethics Board (REB) granted a waiver of the requirement for REB approval of this QI activity.

## 3. Results

Four context, three mechanism and three outcome variables were identified within the data (Table 1). These variables are described in detail below.

### 3.1. Context

The first context variable was the Cancer Centre Size, measured by new patient volumes. Annual new patient volumes ranged from 1273 to 10,935 (Table 2). Half of the RCCs were categorized as small (<3000 patients), while five were medium (between 3000 and 8000 patients) and two were large (between 8000 and 11,000 patients).

The second context variable was the Smoking Cessation Model used (Table 3). OH-CCO recommended the internationally recognized 5As (Ask, Advise, Assess, Assist, Arrange) model as the standard approach to be used by healthcare providers (HCP) [5,7]. Seven RCCs reported using the 5As model. Six used the 3As (Ask, Advise, Act) model [25] and one used a modified 4As (Ask, Advise, Assess, Arrange) model [26].

The third context variable was Smoking Cessation Education (Table 3). Two RCCs integrated cessation education into new staff orientations and three had mandatory in-service training. Five RCCs offered a combination of both. Education was delivered through large or small group presentations. All RCCs offered optional ad hoc education through webinars and rounds.

The fourth context variable was the Referral System (Table 4). Ten RCCs offered both internal and external referral services. Two RCCs offered internal referral services only, and two RCCs offered external referrals only. The timing of the internal referrals varied, but referrals were either arranged for the same day as the patient’s initial appointment, or scheduled at the time of a follow-up appointment. The internal referral provider offered high intensity behavioral counselling and included “quit coaches”, pharmacists, or other trained HCPs. Among the more common external referral services was the telephone quit line operated by the Canadian Cancer Society (Smokers’ Helpline).

### 3.2. Mechanism

The three identified mechanism variables included the Patient Flow Process for Screening, Patient Flow Process for Advising and Referring, and Pharmacotherapy Access, and the first mechanism variable was the Patient Flow Process for Screening (Table 2). Eight RCCs used electronic data systems to capture tobacco screening for smoking status, while five used paper-based systems. One used a paper-electronic hybrid system. In eight RCCs, nurses were primarily responsible for screening patients. In three RCCs, screening was patient-initiated and patients were required to complete a questionnaire independently before speaking with a HCP. In three RCCs, screening was done by clerical staff or health care aides. When screening was conducted by clerical staff, it was at a centralized location, and when it was conducted by non-clerical staff, it was done in clinics.

The second mechanism variable was the Patient Flow Process for Advising and Referring (Table 3). At twelve RCCs, nurses or other frontline staff were responsible for providing advice on the benefits of cessation and for recommending a referral. One RCC had a patient-initiated model using tablet computers, while another relied on clerical staff. None of the RCCs relied on the internal referral providers to advise patients on the benefits of cessation or to recommend a referral.

The third mechanism variable was Pharmacotherapy Access (Table 4). Access to SC pharmacotherapy including recommendations or prescriptions for nicotine replacement therapy (NRT) varied. Ten RCCs routinely recommended pharmacotherapy, with only seven offering it on-site. In those RCCs that discussed pharmacotherapy options, trained internal referral providers were the main prescribers, and pre-printed prescriptions or on-site pharmacists were available to facilitate access. Two RCCs offered financial assistance, including coupons, starter packs or time-limited complimentary NRT to encourage participation in the SC program.

### 3.3. Outcomes

The three outcome variables identified included the: Tobacco Screening Rates, Advised on Benefits and Recommended Rates (Advise Rates) and Accepted Referral Rates. Three small RCCs and one medium had tobacco screening rates above the 70% OH-CCO target, ranging between 73% and 100% (Table 2). Although screening rates were below target in the large RCCs, the overall number of patients screened was relatively high.

Eight RCCs had high advise rates, ranging between 80% and 100%. Four RCCs had high recommend referral rates, ranging between 72% and 83% (Table 3). For accepted referral rates, only five RCCs achieved the target of 20% (Table 4). Across all RCCs, the proportion of referrals that were internal (50%) was higher compared to external referrals (25%) or the use of both types of referrals (25%).

### 3.4. Context–Mechanism–Outcome Linkages

**Figure 1:**
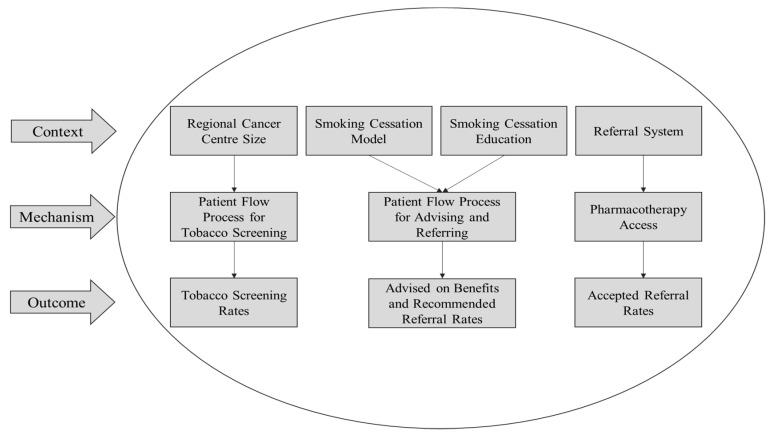
Context–Mechanism–Outcome (CMO) variable interactions. Individual interactions between specific context, mechanism and outcome variables identified based on pattern similarities between top-performing Regional Cancer Centres.

Patterns were observed in the data and were used to construct specific links between context, mechanism and outcome variables (Figure 1). These links were formed to identify similarities in practice between top-performing RCCs. For the first context variable, Cancer Centre Size, four RCCs, three small and one medium, achieved tobacco screening rates that exceeded the target (70%). In the two top performing RCCs, dedicated clerical staff screened patients for smoking status at a centralized location (Table 2).

For the second and third context variables, Smoking Cessation Model and Education, similar patient advised and recommended referral rates were seen in RCCs using the 3As model for smoking cessation (Table 3). Three RCCs using the 3As model—one large, one medium and one small—had the highest patient advice and recommended referral rates in their size categories. These RCCs also offered robust smoking cessation training for staff.

For the fourth context variable, Referral Systems, most RCCs had an internal referral provider located on-site who was able to schedule an appointment or meet with patients immediately following their first visit (Table 4). Tobacco pharmacotherapy was available on-site and recommended or prescribed in the majority of RCCs that had an internal referral provider. Overall, there was a higher rate of internal referral acceptance than external referral acceptance.

## 4. Discussion

There are few reports on implementation of SC interventions in cancer centers. This QI evaluation identified variables that may help to improve implementation of such interventions.

Theoretical links were formed between specific context, mechanism and outcome variables to help explain the varying success at each RCC (Figure 1). It is important to consider that many of the listed variables interact on a broader level as well. For example, referral systems were connected to pharmacotherapy access and the proportion of smokers that accepted a referral. However, this is not to say that referral systems could not influence the patient flow process for tobacco screening and proportion of patients screened for tobacco use to some degree. As such, caution must be taken when interpreting the results.

Our findings show that the two RCCs with the highest Tobacco Screening Rates used a centralized screening process [27] administered by clerical staff, while the remaining twelve RCCs used a decentralized screening process that was either patient-initiated, initiated or run by nurses or health care aides (Table 2). With centralized screening, all new patients were registered and subsequently screened for smoking status at a single location. With decentralized screening [27], patients were admitted to a specific clinic, and were often screened for smoking status during the nursing assessment. The question remains as to whether the high screening rates are due to the centralized screening process, the staffing model, or a combination of both.

While centralized screening may be feasible in small or medium centers where patient volumes are lower, this may present a challenge in larger centers. Large centers wishing to implement a centralized screening process could consider screening patients via telephone, prior to patients’ arrival at the cancer center, where they can then engage with the nurse or clinician. This may help to streamline tobacco screening such that smokers are flagged before they are seen in clinic.

Literature based on the experience of smoking cessations in the emergency department setting has shown that there is a strong need for role clarity and collaborative efforts between HCPs when implementing smoking cessation interventions [28]. Lack of role clarity may lead to duplicated efforts among HCPs and greater inefficiency in delivering the intervention [29,30]. Therefore, when designing a smoking cessation intervention, it is important to ensure that there is role clarity in relation to screening, advising and referring patients to ensure optimal health system efficiency and effectiveness.

Eight RCCs opted to use an electronic data capture system, while five RCCs used a paper-based system. Paper-based systems have been said to be much less efficient than electronic systems as subsequent manual data entry into an electronic database can lead to errors in the data [31]. Interestingly, two RCCs exhibited high screening rates using a paper-based system and screening improved in one of these RCCs after it reverted to a paper-based system. This finding highlights the importance of context and suggests that while the use of information technology continues to grow in the healthcare setting, the concern for common technology-related issues (e.g., network errors and staff unfamiliarity navigating electronic systems) still persist [32]. However, electronic systems are being designed to be much more “paper-like” as they become more user-friendly, inexpensive, and portable [33].

In the three RCCs with the highest Advised on Benefits and Recommended Referral Rates, the 3As Smoking Cessation Model was used and Smoking Cessation Education was offered in the form of in-service staff training and integration into new staff orientation programs. Literature has shown that staff who receive training in SC are more willing to discuss it with patients and refer them to services [34]. Not surprisingly, in the majority of RCCs using the 5As Smoking Cessation Model, there was a drop between Advised on Benefits and Recommended Referral Rates. This finding is reinforced in prior literature that has shown HCPs do not complete all components of the 5As, resulting in fewer patients being referred to a dedicated SC service [35]. In the majority of RCCs, advice and referrals were offered by nurses, which did not seem to have an effect on outcome rates. In only one RCC was the Patient Flow Process for Advising and Referring patient-initiated, resulting in higher advice and recommended referral rates. In this RCC, upfront education about the benefits of SC was provided to cancer patients via tablet computer [36].

While patient-initiated methods for SC education can be effective, having a point of contact with an HCP who is able to reinforce this knowledge is of critical importance [37]. These findings demonstrate that using a briefer 3As model, and implementing a patient flow process that is patient-initiated may be most effective. However, appropriate staff training is also necessary to ensure that SC is discussed further with the HCP.

Findings show that although only five RCCs had Accepted Referral Rates above the target (20%), internal referral acceptance was higher than external referral acceptance (50% versus 25%) and the majority of RCCs had an internal referral provider located on-site. Cancer patients face a high number of clinic appointments during a stressful time when they are likely to be feeling unwell. Dedicated internal referral providers who have adequate training and expertise in SC can offer immediate one-on-one counselling, which may help alleviate this burden from patients [38]. Additionally, they can arrange follow-up with patients to ensure they receive additional cessation support through methods such as telephone counselling. Previous studies have shown that follow-up telephone counselling can double smokers’ quit rates [39,40]. Dedicated counselling and follow-up, which are features of best practice SCIs, have been shown to be reasonably cost-effective [41,42,43]; however, the cost of hiring an internal provider can be a barrier, particularly for large centers where more than one internal referral provider would be needed to meet the demand.

When combined with counselling, SC pharmacotherapy has been found to improve tobacco cessation rates [44]. Ten RCCs discussed, prescribed, recommended or offered pharmacotherapy for cancer patients. The convenience of immediate counselling and low-cost SC pharmacotherapy on-site may improve patient engagement [45,46]. Unfortunately, the literature shows that HCPs may be hesitant to discuss pharmacotherapy with cancer patients if they are not well-educated about appropriate dosing or pharmacodynamics [47,48,49,50]. To respond to this important barrier, many RCCs developed pre-printed prescriptions or allowed pharmacists to prescribe tobacco pharmacotherapy. Nevertheless, educating HCPs about tobacco pharmacotherapy is needed and can help ensure that clinical decision-making continues to be evidence-based [47,48].

### Strengths and Limitations

This QI evaluation has limitations. The SC interventions were assessed by means of self-report by SC champions and there is potential for recall bias. Interviews were captured at one moment in time and may not reflect changes made as interventions evolved. In an effort to minimize this limitation, site visits to each RCC were conducted to learn more about implementation.

## 5. Conclusions

Our findings demonstrate how a realist approach to QI evaluations can be a useful methodology to explore how evidence-based interventions are implemented in practice. We have identified several variables that should be considered when designing and implementing SC interventions in cancer care. For the Patient Flow Process for Screening, the highest performance was seen in RCCs with lower patient volumes that used a centralized screening system administered by dedicated clerical staff. For the Patient Flow Process for Advising and Referring, the highest performance was seen in RCCs that used the 3As model for SC, integrated SC into staff orientations and offered smoking cessation training for staff. For the Referral System, the highest performing RCCs had a robust internal program with a dedicated internal provider and on-site pharmacotherapy access. These observations may be useful to individuals wishing to implement or evolve SC interventions. Future studies will address the clinical effectiveness of the OH-CCO smoking cessation initiative in terms of quit rates and the impact of cessation on cancer treatment outcomes.

## Figures and Tables

**Table 1:** Analytical Frame that anchored interpretation of context, mechanism and outcome variables of the smoking cessation intervention.

	Context	Mechanism	Outcome
Definition	Pre-existing conditions within which programs or interventions are implemented [17,18,19].	The mode of action or available resources that influence the intervention outcomes [17,18,19].	The result of the intervention [18,20].
Variables	(A) Cancer Center Size (i) Small (ii) Medium (iii) Large (B) Smoking Cessation Model (C) Smoking Cessation Education (D) Referral System (i) Internal Referral (ii) External Referral	(A) Patient Flow Process for Tobacco Screening (i) Tobacco Screening Location (ii) Tobacco Screening Contact (iii) Data Capture Platform (B) Patient Flow Process for Advising and Referring (C) Pharmacotherapy Access (i) Recommended or Prescribed (ii) On-Site Availability	(A) Tobacco Screening Rates (B) Advised on Benefits and Recommended Referral Rates (C) Accepted Referral Rates

Note: Small < 3000 patients; Medium 3000–8000 patients; Large 8000–11,000 patients.

**Table 2:** Context and Mechanism variables that predict highest tobacco screening rates.

	Context			Mechanism		Outcome
Regional Cancer Centre	Cancer Centre Size: Patient Volumes	Cancer Centre Size	Patient Flow Process for Tobacco Screening: Location	Patient Flow Process for Tobacco Screening	Patient Flow Process for Tobacco Screening: Electronic or Paper	Tobacco Screening Rates % (*n*)
13	2155 new patients	Small	Centralized screening	Clerical staff	Electronic data capture	99.7% (2148)
4	5914 new patients	Medium	Centralized screening	Clerical staff	Paper data capture	80.6% (4767)
11	2496 new patients	Small	Decentralized screening	Patient-initiated	Paper data capture	80.6% (2011)
8	2808 new patients	Small	Decentralized screening	Nurse	Electronic data capture	73.3% (2058)
14	1273 new patients	Small	Decentralized screening	Nurse	Electronic data capture	67.0% (853)
10	2625 new patients	Small	Decentralized screening	Nurse	Electronic data capture	66.1% (1734)
6	4974 new patients	Medium	Decentralized screening	Nurse	Hybrid data capture	64.9% (3226)
9	2796 new patients	Small	Decentralized screening	Nurse	Electronic data capture	64.9% (1815)
1	10,935 new patients	Large	Decentralized screening	Patient-initiated	Electronic data capture	60.1% (6575)
5	5696 new patients	Medium	Decentralized screening	Health care aide	Paper data capture	60.1% (3424)
3	6669 new patients	Medium	Decentralized screening	Patient-initiated	Paper data capture	58.5% (3901)
12	2185 new patients	Small	Decentralized screening	Nurse	Electronic data capture	57.6% (1258)
7	4284 new patients	Medium	Decentralized screening	Nurse	Paper data capture	52.1% (2234)
2	9643 new patients	Large	Decentralized screening	Nurse	Electronic data capture	44.4% (4285)
Provincial Total						62.5% (40,289)

Note: Provincial targets set for two performance indicators: 70% for new patients screened for tobacco use; 20% for smokers that accepted a referral. Note: Small < 3000 patients; Medium 3000–8000 patients; Large 8000–11,000 patients.

**Table 3:** Context and Mechanism variables that predict highest advise and recommended referral rates.

	Context		Mechanism	Outcome	
Regional Cancer Centre	Smoking Cessation Education	Smoking Cessation Model	Patient Flow Process for Advising and Referring	Advised % (*n*)	Recommended Referral % (*n*)
1	In-Service, Orientation	3As	Patient-Initiated	100% (1059)	81.6% (864)
10	In-Service, Orientation	3As	Nurse	89.6% (310)	73.4% (254)
14	In-Service	5As	Nurse	86.9% (119)	73% (100)
3	In-Service, Orientation	3As	Nurse	83% (639)	82.2% (633)
7	In-Service, Orientation	5As	Nurse	81.1% (292)	66.9% (241)
2	Orientation	5As	Nurse	80.6% (504)	48% (300)
8	Orientation	5As	Nurse	80.4% (336)	44.3% (185)
4	-	5As	Clerical Staff	80.1% (699)	67.7% (590)
6	In-Service	5As	Nurse	70.9% (212)	65.9% (197)
12	Orientation	4As	Nurse	66.5% (131)	45.7% (90)
5	In-Service	3As	Nurse	55.9% (396)	58.1% (412)
9	In-Service, Orientation	3As	Nurse	43.8% (160)	55.3% (202)
13	-	5As	Nurse	16.8% (74)	17.5% (77)
11	In-Service	3As	Nurse	*	21.3% (112)
Provincial Total				69.2% (4931)	59.7% (4257)

* Data not available for patient advised referral.

**Table 4:** Context and Mechanism variables that predict highest accepted referral rates.

	Context		Mechanism		Outcome
Regional Cancer Centre	Referral System: Internal Referral Provider	Referral System: External Referral	Pharmacotherapy Access: Recommendations or Prescriptions	Pharmacotherapy Access: Availability	Accepted Referral Rates % (*n*)
3	Quit Coach	External referral offered	Recommended/prescribed	Not available on site	52.5% (404)
8	None	External referral offered	Not recommended/prescribed	Not available on site	34.7% (145)
9	Quit Coach	External referral offered	Recommended/prescribed	Available on site	23.0% (84)
11	Dental Hygienist	External referral not offered	Recommended/prescribed	Available on site	21.3% (112)
1	Pharmacist	External referral offered	Recommended/prescribed	Available on site	20.1% (213)
7	Support Group	External referral offered	Not recommended/prescribed	Not available on-site	19.7% (71)
5	Pharmacist	External referral offered	Recommended/prescribed	Available on site	18.9% (134)
4	Radiation Therapist	External referral offered	Recommended/prescribed	Available on site	14.4% (126)
13	Social Worker	External referral offered	Not recommended/prescribed	Not available on-site	14.1% (62)
6	Multiple Healthcare Providers	External referral offered	Recommended/prescribed	Available on site	14.0% (42)
2	Psychiatrist, Smoking Cessation Class	External referral offered	Recommended/prescribed	Available on site	13.3% (83)
10	Quit Coach	External referral offered	Recommended/prescribed	Not available on-site	11.0% (38)
12	None	External referral offered	Not recommended/prescribed	Not available on-site	8.1% (16)
14	Nurse Practitioner	External referral not offered	Recommended/prescribed	Not available on-site	7.3% (10)
Provincial Total					21.6% (1540)

Note: Provincial targets set for two performance indicators: 70% for new patients screened for tobacco use; 20% for smokers that accepted a referral.

## Data Availability

The data presented in this manuscript are available on request from the corresponding author. The data are not publicly available due to confidentiality.
